# Information battleground: Conflict perceptions motivate the belief in and sharing of misinformation about the adversary

**DOI:** 10.1371/journal.pone.0282308

**Published:** 2023-03-22

**Authors:** Honorata Mazepus, Mathias Osmudsen, Michael Bang-Petersen, Dimiter Toshkov, Antoaneta Dimitrova

**Affiliations:** 1 Institute of Security and Global Affairs, Leiden University, Leiden, Netherlands; 2 Department of Political Science, Aarhus University, Aarhus, Denmark; 3 Institute of Public Administration, Leiden University, Leiden, Netherlands; Universitat Luzern, SWITZERLAND

## Abstract

Misinformation has emerged as a major societal concern. But why do citizens contribute to the dissemination of falsehoods online? This article investigates this question by focusing on the role of motivated reasoning and, in particular, perceptions of group-based conflict. It examines the effect of perceived conflict on the endorsement of false news in the context of a regional conflict between Russia and the West as experienced by Ukrainian citizens. In our survey experiment, a sample of Ukrainians (N = 1,615) was randomly assigned to read negative false news stories about Russia, the European Union or Tanzania–a country with no stakes in the conflict. The results show that higher perceived conflict between Ukraine and Russia makes Ukrainians less likely to endorse false news targeting the European Union, but more likely to endorse false news that paint a negative picture of Russia. This finding extends the support for motivated reasoning theory beyond Western contexts investigated so far. Importantly, the effects of conflict perceptions remain strong after controlling for group identity and political knowledge of participants. These results advance our understanding of why false information is disseminated and point to the importance of conflict de-escalation to prevent the diffusion of falsehoods.

## Introduction

The circulation of political misinformation and “fake news” on social media has emerged as a major societal concern. But why do citizens contribute to the dissemination of falsehoods? In American politics, scholars increasingly focus on how deepening political divides drive partisans toward uncritical sharing of dubious and often misleading claims about political opponents [1–3]. However, domestic cleavages such as the electoral fight between Democrats and Republicans are not the only conflict lines that may cause falsehoods to spread. Geopolitical conflict between different states is another type of conflict that contributes to the spread of misinformation and false narratives.

Reminiscent of the Cold War, the Russian state has been blamed for weaponizing false news to interfere with elections in America, France, and Great Britain (e.g., [4, 5]) and for flooding Eastern Europe with online fabricated news to drive down support for the European Union and NATO (e.g., [6, 7]). In the context of geopolitical and armed conflicts, the narratives spread by competing state elites can make it difficult for citizens to distinguish truth from falsehoods (see, for example, [8]). In the context of Eastern Europe, digital disinformation campaigns reflect deeper geopolitical cleavages and have intensified the conflict between countries over the years. Nonetheless, we still know relatively little about how experiencing such conflicts affects citizens’ vulnerability to falsehoods. That is, who among the public will be more willing to believe and share false information about actors involved in regional and international conflicts?

We believe the theory of motivated reasoning offers a compelling framework for answering these questions. This theory posits that people are motivated to accept information that supports their preexisting beliefs and, conversely, reject and counter-argue information that challenges those same beliefs [9]. Empirically, this insight has shown substantial promise in explaining how–in the context of, for example, American politics–identities linked to political parties propel citizens to believe and share misinformation, false news, and conspiracy theories that reflect favorably on their political side (e.g., [2, 10]).

In the present manuscript, our first contribution is to examine whether the theory of motivated reasoning extends to explaining beliefs in and engagement with misinformation in Eastern Europe. As we will argue, Eastern Europe differs significantly from the context of American politics, both in terms of its historical legacy with communism, but also regarding the intensity of geopolitical conflict between foreign actors–most notably, the European Union and Russia. How well, we then ask, does the theory help us makes sense of how Eastern Europeans reason about false news about such international actors?

Our second contribution is to identify *who* is most likely to engage in motivated reasoning in the context of this study. The power of political identity in influencing what one believes is not a fixed feature of human psychology; context matters [11]. As conflicts escalate, the gulf between competing groups will naturally widen, making it vital for people to stand fast and shield their political group from attacks by outgroups. On the flip side, harmonious times under which groups peacefully coexist will likely loosen the need to ardently defend the standing of one’s group. This argument, we contend, suggests that *perceptions of group-based conflict* are critical drivers of motivated processing of misinformation. When perceptions of conflict are low, people will hesitate to believe and share misinformation about other groups. When perceptions of conflict run high, people will be much more willing to believe and share misinformation about rival groups.

We test the effects of conflict perceptions on beliefs and intentions to share misinformation among citizens of Ukraine. Located in Eastern Europe, Ukraine has strong historical and cultural ties to Russia. However, following the *Euromaidan* revolution–a wave of pro-European protests culminating with the overthrow of the pro-Russian government and president-elect Viktor Yanukovych—the subsequent annexation of Crimea in 2014, and, most recently, the Russian invasion of Ukraine on 24 February 2022, the relation between Ukraine and Russia has strongly deteriorated. Prior to the invasion, this became readily apparent in Russian efforts at sowing unrest among Ukrainians by spreading propaganda and misinformation (e.g., [12]), perhaps best illustrated by the dissemination of Russian-sponsored false news about the perpetrators of the Malaysian Airlines Flight 17 crash over Ukraine [13]. Ukraine, on its side, has attempted to balance against Russian interference by founding organizations like StopFake.org, which attempts to expose and counter fabricated stories propagated by Kremlin, and bypassing laws restricting Russian broadcasting news. These counter-strategies notwithstanding, Ukrainian public opinion was, at least prior to the recent Russian invasion, characterized by conflicting views about the relationship between Russia and Ukraine, with a large pro-Western/anti-Russian segment of the public and an opposing minority camp who views Russia as a bulwark against “Westernization” and the European Union [14]. Empirically, this divide in views about Russia versus the European Union presents an opportunity for testing whether beliefs and intentions to share false news about hostile actors depend on citizens’ perceptions of conflict.

We test our claims using a survey experiment that randomly assigns a pre-invasion sample of Ukrainians to read negatively framed false news stories about either Russia, the European Union or Tanzania–a country with no stakes in the ongoing regional conflict. Our study was conducted before the Russian invasion of Ukraine on 24 February 2022: the pilot data was collected in 2017 and the main study data in 2020. The findings we report show that the theory of motivated reasoning extends to false news beliefs in Ukraine and thus make clear that the theory has a broad explanatory scope. We show that higher levels of perceived conflict between Ukraine and Russia makes Ukrainian citizens less likely to believe and share false news targeting the European Union, but more likely to endorse false news that paint a negative picture of Russia. Accordingly, our findings speak to current debates about how to counter false news and, especially, the challenges of doing so. As susceptibility to misinformation is rooted in conflict perceptions, it may be difficult to change this susceptibility without dealing with the underlying causes that create an adversarial relationship between the European Union and Russia. In the following sections, we first engage with a broader literature on the psychology of misinformation before presenting our theoretical contributions to this literature. We then present our study and our analysis. We close by discussion the implications of our findings.

### Motivated reasoning and the psychology of false news

“Fake news” can be defined as “fabricated information that mimics news media content in form but not in organizational process or intent” [15]. While this definition may be straightforward, the everyday task confronting citizens of distinguishing between false and true can be daunting. As false news mimics the features of true news, a person browsing the internet for news can rarely tell whether a given story is true or false but will instead have to draw on indirect cues such as source reputation, endorsements by trusted others or simply a gut feeling. Accordingly, we propose that many false news is psychologically equivalent to a broader class of information: *Rumors*. Rumors are typically defined as “claims of fact–about people, groups, events and institutions–that have not been shown to be true, but that move from one person to another and hence have credibility not because direct evidence is known to support them, but because other people seem to believe them” [16] (see also [17]). Rumors acquire power and become socially meaningful because people willingly spread them, both in everyday interactions with family, friends and coworkers, and–more recently–in larger networks on social media.

Why do people believe and share unsubstantiated information like rumors and false news? One view–which also guides prominent efforts to fact-check and inoculate citizens against falsehoods [18, 19]–claims that people want to read, believe and share accurate information but lack the cognitive resources or time to weed out untrue information in favor of true information. This account certainly has merit: Greater levels of sophistication and cognitive reflection have been shown to drive down beliefs in unsubstantiated claims (e.g., [20, 21]. However, when it comes to politics, people may not merely be motivated by the desire to get the facts right.

The theory of *directional motivated reasoning* has become one of the most important approaches for explaining citizens’ political behavior (e.g., [9, 22]). This theory states that rather than carefully evaluating information, people are “biased” information processors who willingly accept information that bolster their worldviews but resist information that contradicts their views. In American politics, the scholarly consensus is now that *political identities*–partisanship, in particular–strongly influence citizens’ reactions to information [1, 2]. Strong partisans will enthusiastically embrace and disseminate stories that affirm their party’s standing–that is, information that either praise the “in-party” for its glorious deeds or accuse the “out-party” for its appalling actions–while brushing aside inconvenient information conflicting with the in-party’s interests. In offline and online political discussions, the reasoning goes, toeing the party line by sharing dubious information that helps one’s party is typically preferred to sharing true information that reflects badly on one’s side. Accordingly, the theory of directional motivated reasoning explains why people often believe and share false news, rumors, conspiracy beliefs, and other types of factually unsubstantiated claims that benefit their in-group (e.g., [3, 10, 17, 23].

However, in our view, two outstanding issues warrant further consideration. The first issue concerns generalizability. Studies examining the effects of motivated reasoning on public opinion are overwhelmingly WEIRD (WEIRD is an acronym for Western, Educated, Industrialized, Rich, and Democratic countries, see [24]), the bulk of studies being conducted in America, relying on representative samples of American citizens. Although the evidence of similar persistent effects of partisan motivated reasoning from other regions of the world is increasing (see, for example, [25] for a field experiment from India showing that interventions to combat false news can achieve opposite effects to the intended ones due to activation of motivated reasoning), we need more evidence from diverse regions to establish the generalizability of motivated reasoning processes.

As historical experiences and political culture often leave imprints on fundamental psychological traits, norms, beliefs, attitudes, and so on, that are relevant for political scientists and psychologists [26, 27], we need to expand the scope of studies to be able to make stronger claims that are representative of the broader human experience. To provide further evidence for the general claims about human psychology, we study the effects of motivated reasoning in a different region with high (geo-)political conflict. This is not to claim that, say, Eastern Europeans process information and misinformation radically different than Americans do. Indeed, in line with motivated reasoning theory, a recent study [28] demonstrated that Ukrainians who identified strongly with their home country were less persuaded by pro-Russian, anti-Western propaganda narratives. However, evidence from a non-Western region with a strong legacy of communism that struggles with democratization and regional conflicts offers an important test case for a theory that makes general claims about human psychology.

A second issue concerns the situations in which motivated reasoning drives misinformation uptake. Group identities–such as partisanship or national identity–may be necessary precursors of motivated information processing. But they are unlikely to tell the whole story. For example, not all loyal Republicans believe false news about Democrats, and even the most devoted Democrats will sometimes reject anti-Republican narratives (e.g., [29]). Accordingly, in the study of Western politics on topics unrelated to misinformation, researchers are increasingly arguing for an approach that emphasizes how contextual factors influence the strength of the link between group identities and motivated reasoning [11]. Many such factors likely exist. But the one most relevant for our purposes concerns perceptions of group conflict [30, 31]. In the next section, we propose that any framework for explaining the effects of motivated reasoning on beliefs in political misinformation must account for the role conflict perceptions can play in driving up or down false news uptake.

### Conflict perceptions and false news endorsement

Misinformation runs rife during periods of elevated threat and group conflict. During the Cold War, many Americans quickly adopted conspiracy theories about the Soviet Union and the alleged subversive activities of communists [20]. In insurgency-affected areas of Thailand and the Philippines, perceptions of threat have led people to adopt “dread” rumors “that forecast feared or calamitous consequences (for example, the other side is winning and when they assume power, they are going to kill us all)” [32]. These examples are not isolated instances. In their study of intergroup violence [33], the authors convincingly demonstrated that “rumors of aggression committed by members of targeted ethnic groups are nearly universal in events that precede deadly ethnic riots.”

Moreover, the *content* of rumors circulated during conflicts is extremely similar across time and space. The content is nearly always extreme and aims to “threaten individuals through their group identities” [34]. These themes also feature prominently in most contemporary political misinformation. In an increasingly polarized political climate in America (e.g., [35]), false news often includes hyper-partisan frames and narratives denigrating the out-party at the expense of the in-party [3, 17]. Similarly, during the 2014 Russo-Ukrainian crisis and the annexation of Crimea, false news promulgated by Russia often featured extreme and hostile narratives about Ukraine, including a dramatic (false) story about the alleged crucifixion of a baby by Ukrainian troops [28].

Why does misinformation about enemy groups spread so rapidly during intergroup conflict? Psychological perspectives highlight the strategic role of rumor dissemination for coordinating group-based actions [36]. During intergroup conflicts, a crucial task is to convince fellow group members to coordinate against the rival group and to persuade third-party actors to join the cause. Hostile rumors about enemy groups are instrumental in such mobilization processes. They provide a “unifying narrative of a terrifying enemy” that allows groups to “close ranks, staunch losses, overcome collective action problems, and sensitize minds to vulnerabilities” [20]. In fact, because rumors and other types of misinformation often contain difficult-to-verify claims about adversary groups and their leaders, they may hold an advantage over true information in coordinating and mobilizing the in-group against perceived out-group enemies [36].

We believe these observations add an important qualifier about when group-centered motivated reasoning becomes activated and affects how people process false news and other hostile rumors. People do not unconditionally believe and share false news consistent with their group identities. The effects will wax or wane, depending on a person’s perceptions of conflict between in- and out-group. The distinction between in-group identity and inter-group conflict perceptions is not purely semantic. People who identify strongly with a social group–be it a political party, an ethnic group or a nation–will often be friendly disposed towards other groups, in which case they have no incentive to believe or share information that can harm them. In fact, the potential harm to one’s social reputation associated with sharing unsubstantiated news that turns out to be false may make them *less* willing to share such information [37]. On the other hand, when conflict perceptions intensify and group identities turn into outright intergroup hostility, motivations to process information in a group-protective manner increase, making people much more likely to believe and share false news that portray rival groups negatively. These predictions about the effects of conflict perceptions on false news endorsement are at the heart of our argument, and it we now set out to test empirically.

### Overview of the present study

We test our arguments in Ukraine. As discussed in the Introduction, Ukraine has deep historical ties with Russia, but a string of recent events–most notably, the Orange Revolution in 2004, the Euromaiden protests, the Russian annexation of Crimea in 2014 and, most recently, the Russian invasion–has trapped the two countries in an intense geopolitical conflict. Even before the February 24, 2022 invasion of Ukraine (our main study took place in December, 2020, see below) Ukraine was considered a battleground state for Russia’s information war against the West. Researchers have carefully detailed how Russian official narratives frame large-scale protests in Ukraine—be it against corruption, against electoral fraud, or for European integration–as Western-supported attacks on the sovereignty and territorial integrity of countries in Russia’s neighborhood [38]. The hostility towards Russia that many Ukrainians now harbor [39] presents a promising opportunity for testing the generalizability of the theory of motivated reasoning.

Our goal is to show that conflict perceptions are a key ingredient in the motivated reasoning processing of false news. Most importantly, our argument has important implications for *which kinds* of false news Ukrainians are likely to find appealing and–as the group-mobilization account implies–most likely to share. We assume–and empirically validate below–that the majority of our sample see themselves as Ukrainians, but that their perception of conflict with Russia nonetheless varies. From this assumption, our model predicts that Ukrainians who believe Russia and Ukraine are trapped in a hostile conflict should refrain from endorsing false news about the European Union–Russia’s main “competitor” in the region–but be more willing to accept false news that portray Russia negatively. Conversely, the willingness to endorse anti-Russian misinformation should be low among Ukrainians with a positive view of the Ukrainian-Russian relationship. Finally, we predict that perceptions of conflict between Russia and Ukraine should not affect the willingness of Ukrainian citizens to believe and share false news about groups of actors not involved in the regional conflict.

### Design

We collected experimental survey data from a representative sample of the Ukrainian population between 18 and 55 years of age living in urban settlements (i.e, settlements with more than 50,000 inhabitants). The online sample was recruited by the local survey agency, Info Sapiens, in December 2020. The survey was administered in Russian and Ukrainian. The sample included 1,615 participants. Of these, 828 were females, the median age was 36 years (min = 18, max = 55), 75% were employed at the time of the study, while 72% had completed a high education. All participants gave informed written consent. The study was conducted in accordance with the principles expressed in the Declaration of Helsinki.

Within the survey, after having read an introductory prompt and given consent to participate in the study, participants answered a series of demographic questions and questions related to their perceptions of conflict between Ukraine, on the one hand, and Russia and the European Union, on the other hand. Next, participants were randomly assigned to one of two treatment conditions or a control condition. In each condition, participants were asked to read three false news story headlines about either Russia, the European Union or, in the control condition, Tanzania. They then indicated whether they believed and wanted to share the stories. Finally, following a series of questions unrelated to the present study, participants were asked to state their identification with Ukraine, Russia and the EU.

### Treatment stories and dependent variables

The research design and the operationalization of the key theoretical constructs presented below were based on a pilot study from 2017, conducted among 586 participants from Belarus, Moldova, and Ukraine. We discuss the details of this pilot study in the S1 File. Briefly, the study showed that perceptions of conflict between the participants’ home countries and Russia predicted the likelihood of believing false rumors about Russia. While these findings support our theoretical expectations, the study suffered from a series of shortcomings in terms of the materials used that challenge the ability to draw firm causal conclusions.

The survey experiment in our main study randomly assigned participants to one of three conditions. Each condition asked participants to read three news story headlines. The story headlines were kept identical across conditions except for the actors involved in the story. As seen in Table 1, the story headlines in the two treatment conditions described how either Russia or the European Union had taken actions that adversely affected Ukrainian citizens–e.g., by testing COVID-19 vaccines on poor Ukrainian villagers, by passing new discriminatory regulations making it easier to fire Ukrainian workers, or by funding violent street gangs roaming the streets of Kharkiv, Ukraine. The control condition asked participants to read the same headlines, except here it was Tanzanian authorities who took aggressive actions towards citizens of Congo. All headlines were false, made up by the authors for the study. For each story, participants were asked to “[i]magine you come across this headline on a news website,” and they were asked to assess on 10-point scales ranging from “not likely at all” to “very likely” whether they believed the headlines and whether they would share them with people in their social networks. Within each condition, participants’ answers to these questions about (1) beliefs in and (2) intentions to share the false news stories were averaged across the three headlines. The resulting two scales comprise the study’s dependent variables and are recoded to range from 0 = *Not likely at all* to 1 = *Very likely*.

**Table 1:** Overview of treatment materials.

Tanzania Condition	Russia Condition	The EU condition
**[TAN1:]** Dangerous testing! The Tanzanian Health Ministry sends medical experts to Congolese villages to test COVID-19 vaccine on socially disadvantaged Congolese. Side effects are unknown.	**[RU1:]** Dangerous testing! The Russian Health Ministry sends medical experts to Ukrainian villages to test COVID-19 vaccine on socially disadvantaged Ukrainians. Side effects are unknown.	**[EU1:]** Dangerous testing! The EU sends medical experts to Ukrainian villages to test COVID-19 vaccine on socially disadvantaged Ukrainians. Side effects are unknown.
**[TAN2:]** Discriminatory Tanzanian law! New Tanzanian regulation will allow firing Congolese employees working in Tanzania. Health and safety rules are their excuse!	**[RU2:]** Discriminatory Russian law! New Russian regulation will allow firing Ukrainian employees working in Russia. Health and safety rules are their excuse!	**[EU2:]** Discriminatory European law! New EU regulation will allow firing Ukrainian employees working in the EU. Health and safety rules are their excuse!
**[TAN3:]** Tanzanian funding linked to street gangs that beat up Congolese in Kinshasa!	**[RU3:]** Russian funding linked to street gangs that beat up Ukrainians in Kharkiv!	**[EU3:]** EU funding linked to street gangs that beat up Ukrainians in Kharkiv!

Why these news story headlines? First, as the headlines were fabricated but presented as information people could find on news websites, they fit the definition of false news introduced earlier. Second, the stories concerned issues (e.g., COVID-19) that were likely salient and meaningful for the participants at the time of the study. Third, in a similar vein, the stories reflected the types of hostile events we expected to be prominent during inter-country conflicts. Further, while our study mainly focuses on the relationship between Ukraine and Russia, the EU headlines were important for testing the context-specificity of conflict perceptions. The annexation of Crimea in 2014 and the subsequent military conflict in the Eastern regions of Ukraine were a direct consequence of citizens’ protests calling for the Ukrainian government to sign the Association Agreement with the European Union. This was the culmination of a long-standing division within the Ukrainian society about choosing a pro-European rather than a pro-Russian course for their country [39]. Accordingly, we expected that higher levels of perceived conflict between Ukraine and Russia would make people readily accept negative false news about Russia, but *less* likely to believe false news painting a negative picture of the EU.

A similar logic guided the decision to include the headlines about Tanzania and Congo in the control condition. To the best of our knowledge, neither of the two countries play an important role in current Eastern European political affairs. For this reason, participants were unlikely to have strong opinions nor great knowledge about Tanzanian-Congolese relations. As such, the control group stories represented a set of “placebo” false news stories: Participants’ decision to believe or share these false stories was not expected to depend on their perceptions of conflict between Ukraine and Russia.

**Fig 1:**
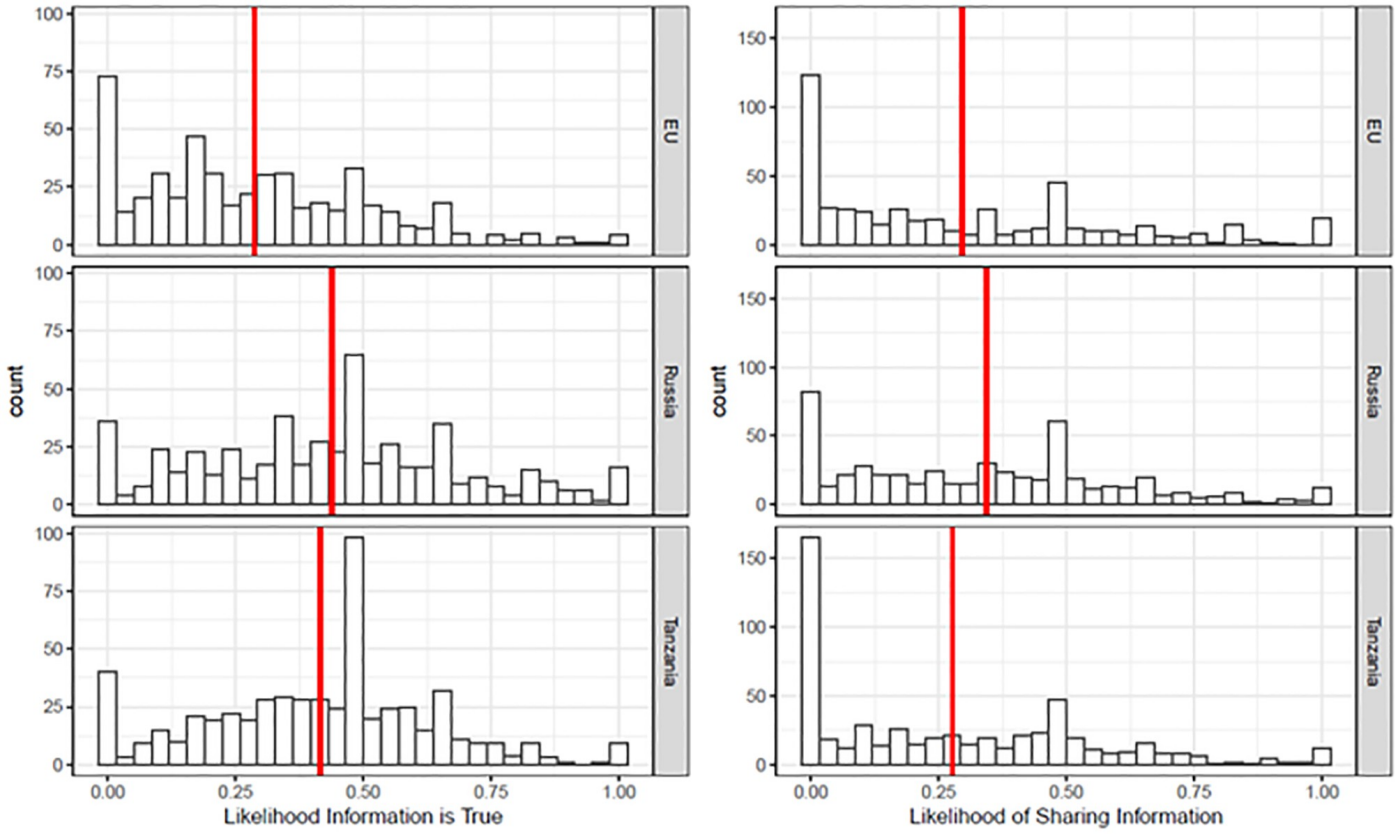
Distribution of beliefs (left) and intentions to share (right) the false news stories by treatment condition: Stories about the EU (top), Russia (middle), and Tanzania (bottom). The red vertical lines give the average value of dependent variables for each condition. The dependent variables range from 0 to 1, with higher values indicating greater beliefs the stories and true and higher intentions to share them.

Fig 1 offers a descriptive summary of the data on participants’ beliefs in and intentions to share the headlines across the three treatment conditions. The left panel of Fig 1 show that participants in the “EU” condition were less likely to believe the false news headlines (*M* = .29, *SD* = .23) compared to participants who read the negative false news about Russia (*M* = .42, *SD* = 26) and Tanzania (*M* = .44, *SD* = .23). The right-hand panel of Fig 1 displays intentions to share the same headlines. Across conditions, sharing intentions were similar and lower than beliefs the stories were true (*M*_EU_ = .30, *SD*_*EU*_ = .29; *M*_*TAN*_ = .28, *SD*_*TAN*_ = .28; *M*_*RUS*_ = .34, *SD*_*RUS*_ = .27). Participants were less likely to share false news about the EU compared to Russia and Tanzanian false news stories were the least likely to be shared. Below, we test–in accordance with our predictions–whether these patterns of belief and sharing are shaped by individual differences in conflict perceptions.

### Independent variables

#### Perceptions of conflict

At the beginning of the survey, participants were asked two questions about their perceptions of conflict between Ukraine and Russia/the EU. Specifically, participants were asked on 10-point scales whether they disagreed or agreed (0 = *Fully Disagree* to 10 = *Fully agree*) with the statements, “Russia [/The European Union] and Ukraine have very different interests; what is good for Russia [/The European Union], is bad for Ukraine”. For analysis, the variables were scaled to range from 0 to 1, with higher values indicating more intense perceptions of conflict. On average, participants viewed the relationship between Ukraine and Russia as substantially more conflictual (*M* = 0.63, *SD* = 0.31) than the relationship between Ukraine and the EU (*M* = 0.42, *SD* = 0.29). Based on our theoretical interests, the main analysis focuses on the measure of Ukraine-Russia conflict perceptions. In addition, we adjust for perceived conflict between Ukraine and the EU in the main models.

#### Covariates

The models presented below adjust for a set of covariates, the most important being *strength of group identity*. Group identities play a decisive role in virtually every study of politically motivated reasoning. Likewise, the strength of in-group identification likely relates to perceptions of intergroup conflict to some extent. Accordingly, to justify our theoretical framework, we must demonstrate that conflict perceptions remain associated with false news uptake *after* accounting for the strength of in-group identity.

Accordingly, our survey included measures of *identification* with the central groups involved in the regional conflict: Ukraine, Russia and the EU. To measure the strength of Ukrainian [/Russian/European] identity, we averaged participants’ agreement with two statements, “I consider myself Ukrainian [/Russian/European]” and “I identify with Ukraine [/Russia/Europe]”. We recoded the scales to range from 0 to 1, with higher values indicating stronger identification with the respective groups. Not surprisingly, our Ukrainian participants identified much more strongly with Ukraine (M = .87, SD = .22) than Russia (M = .20, SD = .27), with European identification (M = .60, SD = .32) falling somewhere in between. Given our sample of Ukrainians, it is unsurprising that the measure of Ukrainian-Russian conflict perceptions correlates positively with Ukrainian identification (*r* = .26) and negatively with Russian identification (*r* = -.37). These correlations are moderately—but not overly–strong. Second, we adjust for *perceptions of power asymmetry*. Uscinski and Parent (2014), and Miller et al. (2016) suggest that group members of the losing side of a power struggle will more readily endorse rumors about enemies perceived to be powerful and threatening. Our framework predicts that conflict perceptions will predict false news uptake even after accounting for the current balance of power and threat perceptions between groups. To measure perceptions of power asymmetries, participants were asked on 10-point scales whether they disagree or agree with the statement, “Russia [/The European Union] is stronger than Ukraine”. We recode the scales to range from 0 to 1, with higher values indicating that Russia/the EU is stronger than Ukraine. Participants generally agreed that Ukraine was weaker than the EU (M = .83, SD = .24) and–to a lesser extent—Russia (M = .64, SD = .32).

Third and finally, we adjust for a series of basic demographic variables–gender, age, level of education and employment status–political interest, and political knowledge. Political knowledge–which plays an important role in some theories about false news endorsement (e.g. Miller et al. 2016)—was measured by summing responses to ten factual questions about politics (e.g.,“Is Ukraine a member of the Eurasian Economic Union?”, “How long is the term of the president of Ukraine?”). The median number of correct responses to the knowledge questions was seven.

## Results

Our key hypothesis is that conflict perceptions affect false news uptake, and that the direction and size of the effect will depend on which actors are depicted in the stories. Empirically, three patterns should emerge. First, and most importantly, as perceptions of conflict between Ukraine and Russia grow stronger, the motivation to believe and share false news about Russia should increase. Logically speaking, one could imagine that the participants both support Russia *and* perceive the Ukrainian-Russian relationship as highly conflictual, which could turn the prediction on its head. Empirically, however, as our sample consists of urban Ukrainian citizens—the majority of whom identify strongly as Ukrainians (see above)–this is not a plausible possibility. For the same reasons, our second expectation is that because of the antagonistic relationship between Russia and the EU–at least as perceived by many Ukrainians–stronger conflict perceptions should drive down endorsement of false news about the EU. Third and finally, as neither Tanzania nor Congo play a role in Eastern European political affairs, perceptions of conflict between Ukraine and Russia should not influence endorsement of these stories.

**Fig 2:**
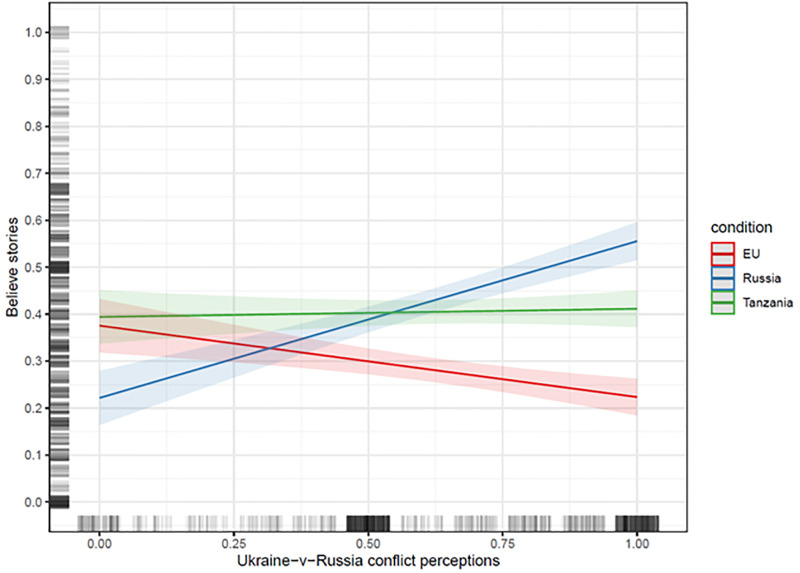
Predicted values of believing the false news stories with 95% confidence intervals by treatment condition -- the EU (red), Russia (blue) and Tanzania (green) -- across the range of conflict perceptions between Ukraine and Russia. Predicted values are based on OLS regression models with the covariates set to their mean (for continuous variables) or median (for categorical variables) values. The vertical and horizontal lines to the left and at the bottom present the jittered distribution of the dependent variable and conflict perceptions, respectively. Conflict perceptions between Ukraine and Russia range from 0 to 1, with higher values indicating stronger perceptions of conflict. The dependent variable ranges from 0 to 1, with higher values indicating stronger beliefs the stories are true. The model adjusts for age, gender, educational level, employment status, political interest, identification with Ukraine/Russia/ the EU, perceptions of power asymmetries, perception of conflict between Russia and the EU, as well as the interactions between Ukraine-Russia conflict perceptions and these covariates.

We examine these predictions by, first, regressing participants’ beliefs in false news on the treatment conditions, perceptions of conflict between Ukraine and Russia, and the interactions between treatments and conflict perceptions. From a causal identification perspective, it is challenging to decide on the “true” model. Accordingly, Table 2 presents results from three regression models that progressively add larger sets of covariates, including the interactions between these covariates and conflict perceptions (see Blackwell and Olson 2021). Because the inclusion of covariates does not substantially alter the main conclusions, we focus on the full model in Column (3). In addition, we present the key results in Fig 2, which is derived from Column (3) and gives the predicted values of believing the headlines for the three treatment conditions, conditional on conflict perceptions between Ukraine and Russia. Here, conflict perceptions and false news beliefs have been scaled to range from 0 to 1.

**Table 2:** Effect of treatment conditions and conflict perceptions on false news beliefs.

	*Dependent variable*:		
	False News Beliefs		
	(1)	(2)	(3)
Russian False News (Rus. FN)	-0.160*** (0.032)	-0.153*** (0.032)	-0.154*** (0.033)
Tanzanian False News (Tan. FN)	0.019 (0.032)	0.020 (0.032)	0.019 (0.032)
Russia-vs-Ukraine Conflict Perceptions	-0.146*** (0.033)	-0.156*** (0.040)	-0.152*** (0.041)
Age		-0.019 (0.015)	-0.024 (0.015)
Education (1 = Low Edu.)		0.002 (0.031)	0.005 (0.031)
Gender (1 = Male)		0.012 (0.028)	0.014 (0.028)
Employment (1 = No work)		-0.022 (0.032)	-0.023 (0.032)
Political Knowledge		0.015 (0.015)	0.010 (0.015)
Political Interest		0.023* (0.014)	0.023 (0.014)
Ukrainian Identification			0.021* (0.012)
Russian Power versus Ukraine			0.023 (0.016)
European Identification			-0.001 (0.015)
Russian Identification			0.002 (0.014)
EU Power versus Ukraine			-0.011 (0.014)
EU-vs-Ukraine Conflict Perceptions			0.018 (0.014)
Rus-vs-Ukr Conflict Perc. X Rus FN	0.496*** (0.046)	0.485*** (0.046)	0.486*** (0.046)
Rus-vs-Ukr Conflict Perc. X Tan FN	0.170*** (0.045)	0.167*** (0.045)	0.169*** (0.046)
Rus-vs-Ukr Conflict Perc. X Age		0.032 (0.021)	0.038* (0.021)
Rus-vs-Ukr Conflict Perc. X Edu.		0.021 (0.044)	0.015 (0.045)
Rus-vs-Ukr Conflict Perc. X Male		0.001 (0.040)	-0.002 (0.040)
Rus-vs-Ukr Conflict Perc. X Empl.		0.027 (0.045)	0.028 (0.045)
Rus-vs-Ukr Conflict Perc. X Pol. Know.		-0.053** (0.021)	-0.047** (0.022)
Rus-vs-Ukr Conflict Perc. X Pol. Int.		-0.004 (0.019)	-0.002 (0.020)
Rus-vs-Ukr Conflict Perc. X Ukr. ID			-0.031 (0.021)
Rus-vs-Ukr Conflict Perc. X Rus. Power vs Ukr			-0.024 (0.022)
Rus-vs-Ukr Conflict Perc. X EU ID			0.001 (0.022)
Rus-vs-Ukr Conflict Perc. X Rus. ID			-0.013 (0.022)
Rus-vs-Ukr Conflict Perc. X EU Power vs Ukr.			0.014 (0.021)
Rus-vs-Ukr Conflict Perc. X EU-vs-Ukr Conflict Perc.			-0.006 (0.019)
Intercept	0.380*** (0.023)	0.379*** (0.028)	0.375*** (0.029)
Observations	1,615	1,605	1,605
Residual Std. Error	0.230 (df = 1609)	0.229 (df = 1587)	0.229 (df = 1575)

**Notes**. Regression coefficients from three models with standard errors in parentheses. Column (1) shows the interaction between treatment conditions and conflict perceptions between Ukraine and Russia on beliefs in the false news headlines without covariates. Column (2) shows the same associations while adjusting for a set of basic covariates while Column (3) adds the full set of covariates. See the “Design” section for operationalizations. To ease interpretation, all continuous covariates have been Z-scored while categorical variables have been coded with their median value as the reference category (“0”). Ukraine-Russia conflict perceptions and false news beliefs have been scaled to range from 0 to 1.

*p <0.1

**p < .05

***p<0.01.

Fig 2 supports our expectations. Consider first the association between conflict perceptions and beliefs in false news about Russia (blue line). As perceptions of conflict between Ukraine and Russia increase from the lowest to the highest level, beliefs in the Russian false news headlines increase by about 33 percentage points, from around .23 to .56 on the 0–1 scale. (In Column (3) of Table 2, this can be derived by taking the absolute difference between the coefficient for “Russia-vs-Ukraine Conflict Perceptions”–b = -0.152 –and the coefficient for the interaction term “Rus-vs-Ukr Conflict Perc. X Rus FN”–b = 0.486. Because the interaction term is statistically significant, we also learn the association between conflict perceptions and beliefs in the fake news headlines differ between the “EU” and “Russia” conditions.) This association, which is statistically significant (p < .001), shows that conflict perceptions strongly predict how Ukrainians view the credulity of information about perceived adversaries. Importantly, results for beliefs in false news about the EU (red line) reveal the opposite pattern. The downward sloping line demonstrates that beliefs in negative false news about the EU drop considerably among participants with strong Ukrainian-Russian conflict perceptions. While this association is weaker than before–moving from the lowest to the highest level of conflict perceptions is associated with about a 15 percentage point decrease in believing the EU false news (p < .001)–it again supports our expectations about the conditionality of conflict perceptions. People seem to keep track of the alliances of actors in conflict situations, and these perceptions about group alignments seem to map into beliefs about information credibility.

Results from the Tanzanian stories strengthen the conclusion about the context-specificity of conflict perceptions. The nearly horizontal green line in Fig 2 demonstrates that conflict perceptions are irrelevant for believing the Tanzanian false news: irrespective of the level of perceived conflict, the predicted belief in the false news headlines hovers around .40 (p > .40). This is an important finding. Conflict perceptions do not motivate people to accept or reject all sorts of unsubstantiated claims. Rather, they matter only for beliefs in false news that are relevant for that specific conflict–here: the conflict between Ukraine and Russia as seen from the perspective of Ukrainian citizens—making people more likely to believe false news targeting their opponents and less likely to believe stories about perceived allies. Accordingly, whether a news story is deemed true or false not only depends on its objective truth-value, which we keep constant her. It also depends on the fit between story content and perceived patterns of conflict.

**Fig 3:**
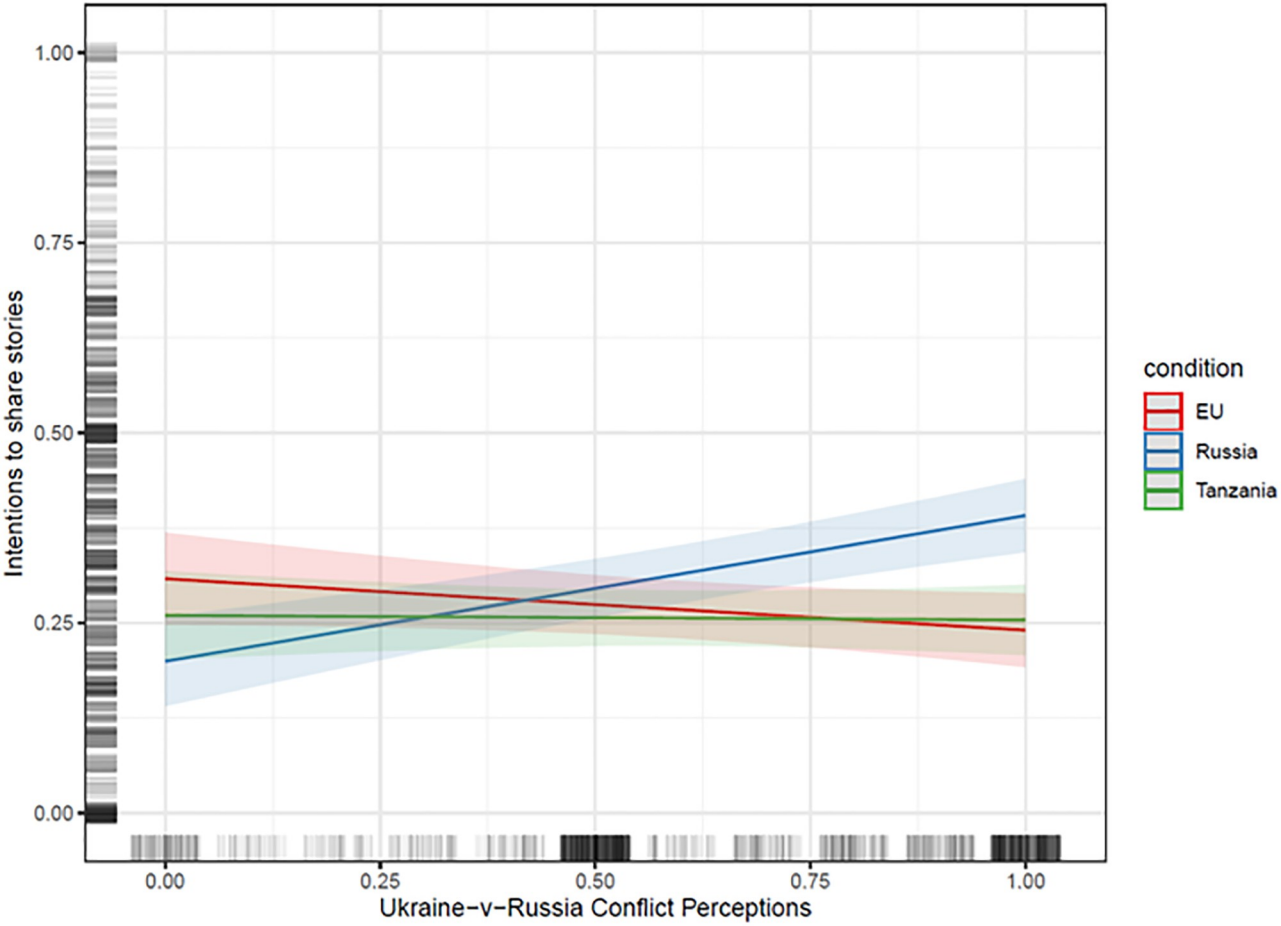
Predicted values of intentions to share the false news stories with 95% confidence intervals by treatment condition -- the EU (red), Russia (blue) and Tanzania (green) -- across the range of conflict perceptions between Ukraine and Russia. Predicted values are based on OLS regression models with the covariates set to their mean (for continuous variables) or median (for categorical variables) values. The vertical and horizontal lines to the left and at the bottom present the jittered distribution of the dependent variable and conflict perceptions, respectively. Conflict perceptions between Ukraine and Russia range from 0 to 1, with higher values indicating stronger perceptions of conflict. The dependent variable ranges from 0 to 1, with higher values indicating stronger intentions to share the stories. The model adjusts for age, gender, educational level, employment status, political interest, identification with Ukraine/Russia/ the EU, perceptions of power asymmetries, perception of conflict between Russia and the EU, as well as the interactions between Ukraine-Russia conflict perceptions and these covariates.

Do perceptions of conflict also condition intentions to share the headlines? To examine this question, we estimate the same regression models except we now switch to sharing intentions as the dependent variable. The results are presented below in Table 3 and Fig 3. Again, we focus on the model in Column (3) which contains the richest set of covariates.

**Table 3:** Effect of treatment conditions and conflict perceptions on intentions to share false news.

	*Dependent variable*:		
	False News Sharing		
	(1)	(2)	(3)
Russian False News (Rus. FN)	-0.162*** (0.038)	-0.152*** (0.037)	-0.148*** (0.037)
Tanzanian False News (Tan. FN)	-0.077** (0.038)	-0.077** (0.037)	-0.081** (0.037)
Russia-vs-Ukraine Conflict Perceptions	-0.123*** (0.039)	-0.118*** (0.046)	-0.087* (0.047)
Age		-0.060*** (0.017)	-0.062*** (0.018)
Education (1 = Low Edu.)		0.056 (0.036)	0.048 (0.036)
Gender (1 = Male)		0.013 (0.032)	0.009 (0.032)
Employment (1 = No work)		0.0004 (0.037)	-0.004 (0.037)
Political Knowledge		0.024 (0.017)	0.021 (0.018)
Political Interest		0.056*** (0.016)	0.055*** (0.016)
Ukrainian Identification			0.007 (0.014)
Russian Power versus Ukraine			0.016 (0.018)
European Identification			-0.002 (0.017)
Russian Identification			-0.007 (0.017)
EU Power versus Ukraine			-0.029* (0.016)
EU-vs-Ukraine Conflict Perceptions			0.025 (0.016)
Rus-vs-Ukr Conflict Perc. X Rus FN	0.331*** (0.054)	0.320*** (0.053)	0.317*** (0.053)
Rus-vs-Ukr Conflict Perc. X Tan FN	0.091* (0.054)	0.088* (0.052)	0.094* (0.052)
Rus-vs-Ukr Conflict Perc. X Age		0.061*** (0.024)	0.071*** (0.024)
Rus-vs-Ukr Conflict Perc. X Edu.		-0.005 (0.051)	-0.010 (0.051)
Rus-vs-Ukr Conflict Perc. X Male		0.014 (0.046)	0.021 (0.046)
Rus-vs-Ukr Conflict Perc. X Empl.		0.010 (0.052)	0.016 (0.052)
Rus-vs-Ukr Conflict Perc. X Pol. Know.		-0.110*** (0.025)	-0.098*** (0.025)
Rus-vs-Ukr Conflict Perc. X Pol. Int.		-0.016 (0.022)	-0.011 (0.023)
Rus-vs-Ukr Conflict Perc. X Ukr. ID			-0.022 (0.024)
Rus-vs-Ukr Conflict Perc. X Rus. Power			-0.041* (0.025)
Rus-vs-Ukr Conflict Perc. X EU ID			-0.002 (0.025)
Rus-vs-Ukr Conflict Perc. X Rus. ID			0.036 (0.025)
Rus-vs-Ukr Conflict Perc. X EU Power			0.021 (0.024)
Rus-vs-Ukr Conflict Perc. X EU-vs-Ukr Conflict Perc.			-0.003 (0.022)
Intercept	0.375*** (0.027)	0.348*** (0.032)	0.334*** (0.033)
Observations	1,615	1,605	1,605
Residual Std. Error	0.273 (df = 1609)	0.264 (df = 1587)	0.262 (df = 1575)

**Notes**. Regression coefficients from three models with standard errors in parentheses. Column (1) shows the interaction between treatment conditions and conflict perceptions between Ukraine and Russia on intentions to share the false news headlines without covariates. Column (2) shows the same associations while adjusting for a set of basic covariates while Column (3) adds the full set of covariates. See the “Design” section for operationalizations. To ease interpretation, all continuous covariates have been Z-scored while categorical variables have been coded with their median value as the reference category (“0”). Ukraine-Russia conflict perceptions and false news beliefs have been scaled to range from 0 to 1.

*p <0.1

**p < .05

***p<0.01.

The results are similar to those presented above. While the associations are generally weaker–which is unsurprising given the smaller average treatment effects on sharing intentions–we observe a pattern in which higher Ukrainian-Russian conflict perceptions are associated with increased intentions to share anti-Russian false news (Average Marginal Effect (AME) = .23, p < .001) but decreased intentions to share the EU false news (AME = -.09, p = .06). While the latter finding is statistically insignificant at the conventional level, the overall picture is clear: conflict perceptions not only turn up or down beliefs in false news about perceived enemies and allies, they also condition intentions to share those same stories. As false news in the digital world gain power in part because people willingly share them with friends and followers on social media, this is an important finding that we return to in the Discussion. Additionally, conflict perceptions do not condition the impact of the conflict-irrelevant false news stories about Tanzania (AME = .01, p > .60). This again is consistent with our theory.

Finally, to test the generalizability of this pattern, we ask: Do perceptions of conflict between Ukraine and the EU (rather than Russia) also affect the willingness to believe and share the headlines? As we show in the analyses in the Supplemental Materials (SM2a Table in S1 File), participants who view the Ukrainian-European relationship as conflictual are much more inclined to believe and share anti-EU false news narratives but are less willing to endorse Russian false news. These results too align with the theory showing that conflict perceptions are a mechanism that increases the acceptance of false news across different perceived adversaries.

### Group identity, power asymmetries, and knowledge

**Fig 4:**
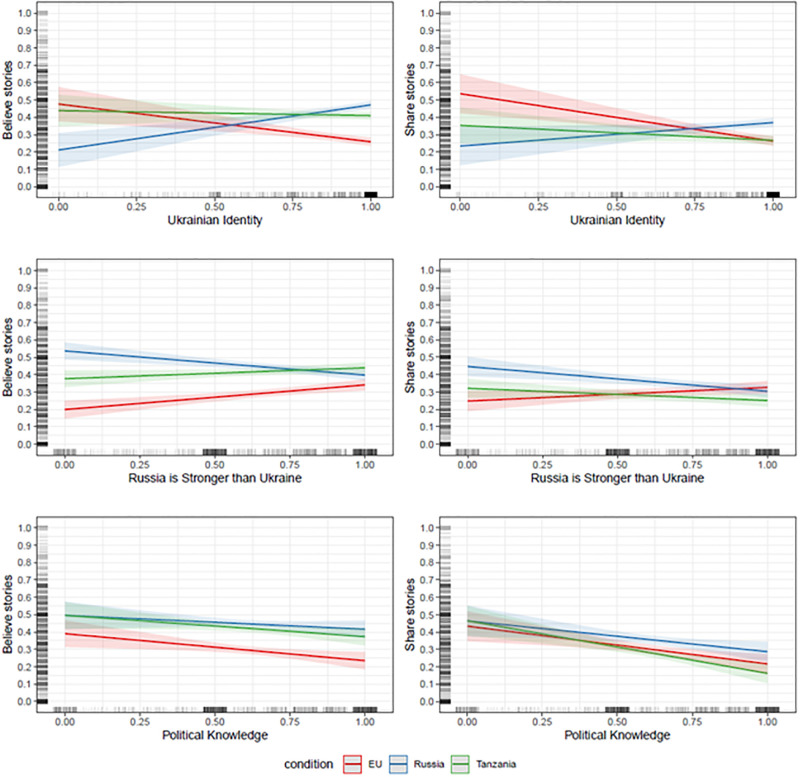
Predicted values of believing and intentions to share the false news with 95% confidence intervals by treatment condition and across the range of Ukrainian identity (upper panels), perceptions of power asymmetry (middle panels) and political knowledge (lower panels). Predicted values are based on OLS regression models. Ukrainian Identity, perceptions of power asymmetry and political knowledge range from 0 to 1. The dependent variables range from 0 to 1, with higher values indicating stronger beliefs the stories are true and stronger intentions of sharing the news stories. The models adjust for age, gender, educational level, employment status, political interest and perceptions of conflict between Russia and Ukraine as well as the interactions between treatment conditions and the covariates.

The results presented above show that conflict perceptions predict false news endorsement *after* adjusting for participants’ national identity, political knowledge, and perceptions of power asymmetries between Ukraine and Russia–variables that earlier studies have highlighted in analyses of false news uptake. Our key finding, however, should not be taken to mean that these other factors are irrelevant for people’s decisions to accept false news. Fig 4, shown below, is based on the same type of regression models used to construct Figs 2 and 3. It shows how beliefs (left-hand panels) and intentions to share (right-hand panels) false news depend on national identity, political knowledge, and perceptions of power after adjusting for the other covariates (see SM2b-SM2d Table in S1 File for regression tables).

The upper panels of Fig 4 reveal results that fully align with a group-oriented motivated reasoning account wherein people believe and share information congruent with their group identity. Thus, participants with a strong Ukrainian identity are more likely to believe (AME = .26, p < .001) and share (AME = .14, p < .01) the anti-Russian false news than participants with a weak identity. In contrast, participants who identify with Ukraine are less likely to believe (AME = -.22, p < .001) and share (AME = -.28, p < .001) false news about the EU. Ukrainian identity, finally, is not associated with beliefs and intentions to share false news about Tanzania.

Turning to Fig 4‘s middle panel, we find evidence that perceptions of power asymmetries between Russia and Ukraine also relate to false news endorsement, but not in the expected direction. As can be seen, Ukrainians who perceive Russia to be stronger than Ukraine are *less* likely to believe (AME = .14, p < .001) and share (AME = -.14, p < .001) anti-Russian false news but *more* likely to believe (AME = .14, p < .001)) and share (.08, p = .06) negative headlines about the EU. These findings do not support the claim that people mostly endorse false news about competing groups when these groups are perceived to be powerful and threatening.

Further, the lower panels show that while political knowledge does not condition the treatment effects, participants with higher levels of political knowledge are generally less likely to endorse false news. For beliefs in false news, the association is only modest. Across conditions, moving from the lowest to the highest level of political knowledge is associated with an 8–16 percentage point drop in false news beliefs (*p’s* between .01-.20). In contrast, we observe a much stronger and negative association between political knowledge and intentions to share false news. In all conditions, participants with the highest level of knowledge are about 17–30 percentage point less likely to indicate they will share false news (*p’s* < .001). Insofar political knowledge reflects concerns over whether information is true or false, the results suggest that beliefs and decisions to share not only rest on group-fueled motivations. Accuracy motivations matter too.

Taken together, these results show that factors emphasized in earlier work do influence the belief in and sharing of false news (although, in the case of perceptions of power asymmetries, not in the expected direction). Still, these findings do not take away the main conclusion of this investigation: receptivity to misinformation varies predictably according to how well the information content fits with a person’s perceptions of conflict. In this way, our results add important new theoretical insights into who are more or less susceptible to the influence of false news. Directional processing of information is not just a function of the strength of group identity; it in large part depends on *inter*group dynamics and perceptions of whether group relationships are peaceful or antagonistic.

## Conclusion

Eastern Europe has emerged as a key battleground for misinformation campaigns related to the increasing conflict between Russia, on the one hand, and NATO and the European Union, on the other hand. For these reasons, it is of outmost importance to examine how citizens in Eastern Europe respond to false news. The particular context of Eastern Europe allows us to get a fuller picture of the factors that shape susceptibility to this type of misinformation. To identify these factors, our manuscript integrates theories of motivated reasoning into the study of false news endorsement and extends prior studies to more precisely identify *who* is most likely to engage in the motivated processing of false news.

Our findings demonstrate that perceptions of conflict between Ukraine and Russia are a major predictor of false news uptake among our sample of Ukrainian participants. Ukrainians who perceived Russia and their home-country’s interests to clash were likely to endorse false news that denigrates Russia but rejected anti-European false news narratives. While previous studies have emphasized the role of group identities in igniting motivated reasoning processes, the findings we present show that conflict perceptions operate over and beyond the strength of national identity. Group identities, in other words, become a stronger motivational force when linked to perceptions of inter-group conflict.

To be sure, the present study is not without limitations. Our results rest on observational measures of conflict perceptions, and the usual (but important) caveats regarding causal identification applies here. Though we did take steps to strengthen our ability to cast our findings in causal terms–e.g., by adjusting for a series of covariates that earlier research highlights as important–we cannot definitively rule out missing confounders. The ideal study would experimentally induce higher or lower levels of conflict perceptions and track the downstream effects on false news beliefs. However, such an experimental study would be both practically and ethically challenging. Another limitation of our study relates to the sample of participants. We have collected a sample of urban population from the regions under the control of the Ukrainian government at the time of data collection. It could be that the relationship between conflict perception and belief in false news works differently for people living in rural areas, given their on average lower level of online information consumption. Future work could test if the identified relationship generalizes to this population.

These caveats notwithstanding, we believe that the present findings have important implications. They demonstrate that theories of motivated reasoning can successfully be applied to understand the psychology of those exposed to misinformation campaigns in Eastern Europe and, potentially, beyond. In this way, our study supplements a string of recent studies demonstrating the generalizability of motivated reasoning theory for understanding false news uptake [25, 32]. Furthermore, we show that conflict perceptions–and not just group identities or perceptions of power—are crucial for false news uptake. Furthermore, our findings inform discussions about how to counter the effects of misinformation campaigns and the circulation of false news. False news becomes powerful only to the extent people believe the stories they read and want to share them with others.

Reducing conflict perceptions, then, could constrain processes of motivated reasoning and help limit the circulation of and exposure to false news online and on social media in particular. While appealing, this approach comes with an inherent limitation. Conflict perceptions do not appear out of thin air, but often stem from hard-to-change societal and geopolitical divides. This observation appears particularly pertinent given the military escalation in the wake of the Russian invasion of Ukraine. At the time of writing, a peaceful resolution to the conflict seems distant, with no obvious signs that attempts at de-escalation align with the current interests of the actors involved. In fact, the conflict has made clear that false news and other forms of misinformation often serve to justify aggressive actions, especially on the Russian side. As long as the two sides remain locked in a core geopolitical struggle, perceptions of conflict will remain high, and attempts to limit the circulation and impact of misinformation through corrections and other means will likely continue to be ineffective. As a result, the de-escalation of societal divides might be a more general guiding principle for groups and institutions hoping to prevent misinformation from spreading.

## Supporting information

S1 File (PDF)
