# Correction: Information battleground: Conflict perceptions motivate the belief in and sharing of misinformation about the adversary

**DOI:** 10.1371/journal.pone.0302621

**Published:** 2024-04-18

**Authors:** Honorata Mazepus, Mathias Osmundsen, Michael Bang Petersen, Dimiter Toshkov, Antoaneta Dimitrova

The second and third author’s name are spelled incorrectly. The correct name is: Mathias Osmundsen and Michael Bang Petersen, respectively. The correct citation is: Mazepus H, Osmundsen M, Bang Petersen M, Toshkov D, Dimitrova A (2023) Information battleground: Conflict perceptions motivate the belief in and sharing of misinformation about the adversary. PLoS ONE 18(3): e0282308. https://doi.org/10.1371/journal.pone.0282308.
