# Supplementary material for: Information battleground: Conflict perceptions motivate the belief in and sharing of misinformation about the adversary
Source: PLoS One. 2023 Mar 22;18(3):e0282308. doi: 10.1371/journal.pone.0282308 (PMC10032542; doi:10.1371/journal.pone.0282308)
Supplement: S1 File — (PDF) [file pone.0282308.s001.pdf]

Supplemental Materials

Our data come from a pilot study and a main study. The pilot study was a pen-and-paper survey administered to around 600 participants from Belarus, Moldova, and Ukraine in 2017. It served as an informal pre-registration for the main study. Its purpose was to develop and test measures of the key theoretical constructs in a geographical context similar to the main study (i.e., Eastern Europe). The main study is discussed at length in the main text. Below, we elaborate on the study design and offer additional analyses.

1. Pilot Study

1a. Sample Characteristics and question wordings for key variables

Procedure. The pilot study examined beliefs in fake news about Russia and the European Union among citizens of Belarus, Moldova, and – as in the main study – Ukraine. All three countries are former republics of the Soviet Union, and observers have highlighted Russia's recent interest in reclaiming its sphere of influence over them (Fredheim, 2015; Oates, 2016). In this way, the three countries offer a good first opportunity for exploring whether inter-group conflict perceptions predict fake news beliefs about perceived antagonistic actors.

Sample characteristics. The pilot study was conducted with the help of local partner institutions (SYMPA in Belarus, IDIS 'Viitorul' in Moldova, and UIPP in Ukraine). We used non-probability sampling to collect data from a diverse pool of respondents in different regions and locations. The Belorussian sample (n = 210) was collected in four cities (Minsk, Grodno, Gomiel, and Vilnius[1]), and surroundings of Minsk; the Moldovan sample (n = 183) was collected in seven districts (Chișinău, Ialoveni, Orhei, Comrat, Călărași, Cimișlia, and Anenii Noi); and the Ukrainian sample (n = 193) was collected mainly in five cities in the Odesa region, in the Kyiv region, and small samples in the Chernikhiv and Poltava regions. Participants were presented with an instruction and a consent form (printed out or read out to them) and took part in the survey after expressing consent. They were informed that participation is voluntary, that they can stop participation at any moment without consequences, and that the data will be treated as confidential and used only

[1] In Vilnius, we used a sample of Belorussian students who study at the European Humanities University – a private, non-profit liberal arts university founded in Minsk and, due to forced closure by the Belorussian authorities in 2004, relocated to Vilnius.

for the purpose of research.

In Belarus, 51% of participants were female and the average age was 37 years old (SD = 13). In Moldova, 72% of participants were female and the average age was 42 years old (SD = 15). In Ukraine, 60% of participants were female and the mean age was 39 years old (SD = 13).

Key survey measures.

Perceptions of inter-group conflict: Our key explanatory variable is perceptions of conflict between Russia and the participant's home country. To measure this construct, participants were asked whether they agreed or disagreed with the statement: "Russia and Ukraine [Belarus/Moldova] have very different interests; what is good for Russia, is bad for Ukraine [Belarus/Moldova]". Answers were measured on a ten-point scale ranging from "Fully disagree" to "Fully agree", which was subsequently scaled to range from 0 to 1, with higher values indicating higher perceptions of conflict (M_{BEL} = .47, SD_{BEL} = .31; M_{MLD} = .56, SD_{MLD} = .32; M_{UA} = .59, SD_{UA} = .31). The mean values of the variables make clear that perceptions of conflict versus Russia were moderately high in the three countries, especially in Ukraine, but, as the standard deviations show, large variation around these mean values exists.

Fake News beliefs: We measured participants' beliefs in fake news about Russia and the European Union. The stories were chosen by the study authors from a list of fake news compiled by country experts and fact-checking sites. Three criteria determined the selection of stories. First, to increase the study's external and ecological validity, the stories had to be shared widely online prior to data collection. Second, to fit our definition of fake news, the stories had to be evaluated and flagged as "false" by fact-checkers. Third, the stories had to speak to an ongoing conflict between Russia or the European Union, on the one hand, and the participant's home country, on the other.

Consequently, for the *anti-Russian fake news*, participants in both Ukraine and Belarus read a headline stating that "Europe officially recognized Russia as a sponsor of international terrorism." Meanwhile, Moldovan participants read a fake news headline stating that "Russia supports the establishment of a new republic in the northern part of Moldova". For the *fake news about the European Union*, Ukrainian participants read the headline "Europe accused Ukraine of increasing radiation". Belorussian participants were presented with the headline that "Neighboring EU members, Poland and Lithuania, funded military training of protesters in Belarus", while Moldovan participants read that "Moldovan exports to the European Union dropped dramatically after the

conclusion of the Association Agreement". Participants were told to carefully read and to indicate on three-point scales whether they believed the headlines (1 = Yes, 2 = No, 3 = I'm not sure).

Additional covariates: In addition to the key variables of interest, the regression analyses presented below included, first, a measure of conflict perceptions between Russia and the European Union. This was included to rule out that a (potential) association between perceptions of conflict between participants' home country and Russia was not simply driven by a general predisposition to view the world in terms of conflict (Q:"Russia and the European Union have very different interests; what is good for Russia, is bad for the European Union"; A: 0 = Fully Disagree; 10 = Fully Agree). Second, we included a measure of perceived Russian power versus participants' home country (Q: "Russia is stronger than BEL/MLD/UA"; A: 0 = Fully Disagree; 10 = Fully Agree). Third, the analyses adjusted for a set of sociodemograhpic variables – gender, age, education – and, because the main analyses pool data across countries, two country dummies.

1b. Analysis

Are perceptions of conflict associated with belief in fake news? Our central prediction is that conflict perceptions increase belief in fake news that negatively portray enemy groups. To test this prediction, **Table SM1** gives results from ordered logistic models that regress fake news beliefs about Russia (column 1) and the EU (column 2) on perceptions of conflict between Russia and the participant's home country. The models adjust for a set of sociodemographic covariates (gender, age, education), perceptions of conflict between the participant's home country and the EU, and the perceived power of Russia. The models pool participants across countries, but include country indicators.

Consistent with our argument that fake news beliefs emerge from conflict perceptions, Model 1 shows a positive association between perceptions of conflict with Russia and beliefs in anti-Russian fake news ($b = 1.64$, $p < .001$). In contrast, but still in line with our argument, Model 2 shows a negative relationship between Russian conflict perceptions and beliefs in fake news targeting the EU ($b = -1.26$, $p < .001$).[2] Accordingly, likely due to the antagonistic relationship between Russia and the EU, individuals who oppose Russia are less likely to believe fake news that portray the EU

[2]Additional analyses show that these associations between conflict perceptions and belief in fake news are generally similar across the three countries. We examined this by testing interactions between the conflict perceptions and country indicators. Only in one case do we observe a significant interaction. Thus, the fake news targeting Russia, the association between beliefs and conflict perceptions are stronger in Belarus than in Ukraine (p=.012). However, even in Ukraine, the association has the predicted sign and is marginally close to significant ($b = 1.01$, $p = .12$).

negatively.

Ordered logistic regression coefficients can be difficult to interpret. Accordingly, **Fig SM1** plots the predicted values from Model 1 and 2. As can be seen from these models, the strongest associations are for the correct assessment that the news story is “False.” Among those who do not see any conflict between their home country and Russia (equaling a value of 0 on the x-axis), the probability of identifying a fake news story targeting Russia as false is slightly above 60%. Among those who see maximal conflict (equaling a value of 1), the probability of a correct assessment is as low as 20%. In the case of fake news targeting the EU, these numbers are about 40% and 60%, respectively. In both cases, whether or not a news story is identified as true or false seemingly depends not just on its (constant) objective truth value but also on how the story aligns itself with perceived patterns of conflict.

Importantly, the associations of perceptions of conflict between Russia and the home country adjust for two key theoretical variables. First, the associations in Models 1 and 2 adjust for general conflict perceptions by including perceptions of conflict between Russia and the EU. For both fake news targeting Russia and the EU, respectively, the associations between fake news belief and general conflict perceptions are non-significant (see Table 1, Models 1 and 2). In this way, the associations just documented do not reflect that people who are predisposed to view the world in terms of conflict are more likely to believe any kind of fake news. It is specifically conflicts that one has a stake in (here, conflict between Russia and one’s home country) that increase belief in fake news, and only if these fake news targets the antagonist. Second, the associations are adjusted for perceptions of Russia’s power. For fake news targeting Russia and the EU, respectively, the associations between belief in fake news and the perceived power of Russia are insignificant (see Table 1, Models 1 and 2). This suggests that fake news belief is unrelated to general perceptions of threat and power asymmetries. Instead, the driver of such beliefs is perceptions of conflict, no matter whether or not one’s country is the more or less powerful than its opponent.

Together, the results reported in Table 1 and Figure 1 suggest that people believe in fake news stories to the extent that (1) they perceive the target of the news story as involved in a conflict and (2) the framing of the news story resonates with their own side-taking in this is conflict. Hence, East Europeans who see their country involved in a conflict with Russia believe fake news stories that target Russia and reject fake news stories that target the EU, i.e., the ’enemy’ of their ’enemy’.

	Fake News: Russia	Fake News: The EU
Perception of Conflict with Russia	1.645***	−1.259***
	(0.361)	(0.367)
Perceptions of Conflict btw Rus and the RU	−0.453	0.621
	(0.385)	(0.404)
Perceived Russian Power	−0.112	−0.066
	(0.310)	(0.315)
Female	0.311*	0.142
	(0.178)	(0.183)
Age	−0.006	0.017**
	(0.006)	(0.007)
Education	−0.567	−1.023**
	(0.396)	(0.416)
Ukraine	0.564**	0.373
	(0.223)	(0.231)
Moldova	0.468**	0.911***
	(0.218)	(0.225)
Cut 1 (constant)	−0.036	−0.038
	(0.524)	(0.546)
Cut 2 (constant)	1.988***	2.225***
	(0.532)	(0.558)
Observations	519	519

Table SM1. Associations Between Predispositions and Belief in Fake News about Russia and the EU. Entries are ordered logistic regression coefficients with standard errors in parentheses. All variables are scaled between 0 and 1, except age which is in years. The dependent variable, belief in the news story, is scaled as 0 (The news story is fake), .5 (Not sure) and 1 (The news story is true). For the country variable, the reference category is "Belarus". * $p < 0.10$, ** $p < 0.05$, *** $p < 0.01$.

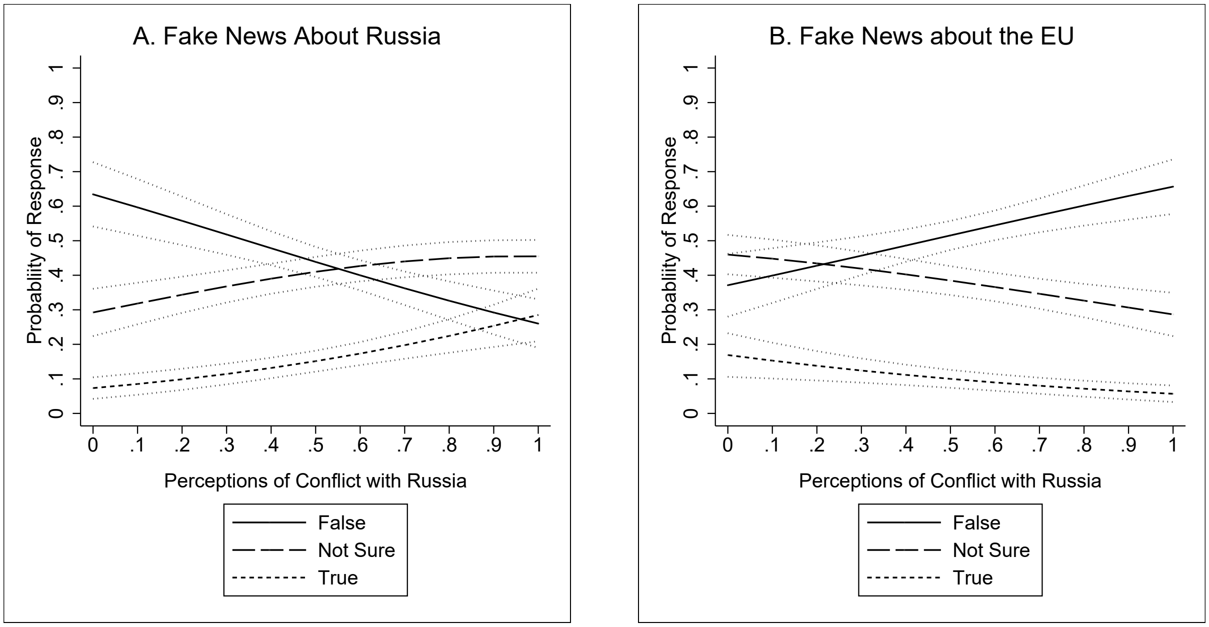

Fig SM1. Association Between Perceptions of Conflict and Fake News Beliefs. Panels are estimated based on models from Table SM1.

2. Main Study

2a. Sample Characteristics and question wordings for key variables

Sample characteristics. We refer to the main text for a discussion of the sampling strategy and the characteristics of the study participants.

Key survey measures.

Perceived conflict between Ukraine and Russia [/the EU]: Please answer the questions below in line with your opinion: (0 = Fully disagree; 5 = Neutral; 10 = Fully agree)

- Russia and Ukraine have very different interests; what is good for Russia, is bad for Ukraine
- The European Union and Ukraine have very different interests; what is good for the European Union, is bad for Ukraine

Perceived power asymmetry between Ukraine and Russia [/the EU]: Please answer the questions below in line with your opinion: (0 = Fully disagree; 5 = Neutral; 10 = Fully agree)

- Russia is stronger than Ukraine
- The European Union is stronger than Ukraine.

Identification with Ukraine [/the EU/Russia]: To what extent do you agree with the following statements?: (0 = Fully disagree; 5 = Neutral; 10 = Fully agree)

- I consider myself Ukrainian/European/Russian
- Q40. I identify myself with Ukraine/Europe/Russia.

Political Interest: How interested would you say you are in politics (1=Not at all interested, 7 = Very interested)

Political Knowledge: of correct replies

- Is Ukraine a member of the Eurasian Economic Union? A: 1- Yes, 2- No
- Who is the current Prime Minister of Ukraine? A: 1- Arseniy Yatsenyuk, 2- Denys Shmyhal, 3- Yulia Tymoshenko, 4- Oleksandr Turchynov
- Who elects mayors of cities in Ukraine A: 1- The President, 2- Citizens of the city by popular vote, 3- Verkhovna Rada, 4- City Council
- Do people vote for bureaucrats in elections? A: 1- Yes, 2- No

- Which one of these institutions is a part of public administration: A: 1-United Service Centre, 2- Parliament ; 3- Schools, 4- Prime-minister
- How long is the term of the president of Ukraine? A: 1- 3 years, 2- 4 years, 3- 5 years, 4- 6 years
- Who is the current Minister of Foreign Affairs of Russia? A: 1- Dmitry Medvedev, 2- Alexei Kudrin, 3- Vladimir Putin, 4- Sergey Lavrov
- Which country is a permanent member of the Security Council of the United Nations? 1- Poland, 2- Russia, 3- Ukraine, 4- Lithuania
- Is Ukraine a member state of the European Union? A: 1- Yes, 2- No
- Who is the current President of the European Commission? 1- Ursula von der Leyen, 2- Donald Tusk, 3- Charles Michel, 4- José Manuel Barroso

Fake News headlines - Tanzania: Imagine you come across this headline on a news website:

- Dangerous testing! The Tanzanian Health Ministry sends medical experts to Congolese villages to test COVID-19 vaccine on socially disadvantaged Congolese. Side effects are unknown
- Discriminatory Tanzanian law! New Tanzanian regulation will allow firing Congolese employees working in Tanzania. Health and safety rules are their excuse!
- Tanzanian funding linked to street gangs that beat up Congolese in Kinshasa!

Fake News headlines - Russia: Imagine you come across this headline on a news website:

- Dangerous testing! The Russian Health Ministry sends medical experts to Ukrainian villages to test COVID-19 vaccine on socially disadvantaged Ukrainians. Side effects are unknown.
- Discriminatory Russian law! New Russian regulation will allow firing Ukrainian employees working in Russia. Health and safety rules are their excuse!
- Russian funding linked to street gangs that beat up Ukrainians in Kharkiv!

Fake News headlines - the EU: Imagine you come across this headline on a news website:

- Dangerous testing! The EU sends medical experts to Ukrainian villages to test COVID-19 vaccine on socially disadvantaged Ukrainians. Side effects are unknown.
- Discriminatory European law! New EU regulation will allow firing Ukrainian employees working in the EU. Health and safety rules are their excuse!
- EU funding linked to street gangs that beat up Ukrainians in Kharkiv!

Dependent variables - Fake News headlines (same for all conditions):

- In your view, how likely is it that this information is true? Please indicate your answer on the scale from 0-not likely at all to 10-very likely A: 0 = not likely at all, 10 = very likely

- How likely would you be to share this information in your social circle? A: 0= not likely at all, 10 = very likely
- Do you recall seeing this reported or discussed in the media (including the Internet and social networks)? A: 0 = No, 1 = Yes

In the main analyses, we use as our dependent variables participants' answers to the first two questions about whether they believe the information and whether they want to share the information.

2b. Analyses

Table **SM2a** shows regression models examining how perceptions of conflict between Ukaraine and the EU condition the treatment effects on fake news endorsement. The "Russian Fake News" and '´Tanzanian Fake News" entries er indicators for the treatment conditions, with the "EU Fake News" serving as the baseline condition. "EU-vs-Ukraine Conflict Perceptions" captures participants' perceptions of conflict between Ukraine and the EU. The models adjust for the same covariates as in the "full models" presented in the main text, except here we now interact these covariates with "EU-vs-Ukraine Conflict Perceptions". In the table – and the ones that follow – all covariates have been Z-scored while categorical variables have been coded with the median value as the reference category. The key variables of interest – here: "EU-vs-Ukraine Conflict Perceptions" – and the measures of fake news endorsement have been scaled to range between 0 and 1.

Tables **SM2b-d** give model results used to construct Figure 4 in the main text. The models follow a similar pattern in which the key independent variable – Ukraininian identification, perceptions of power asymmetry between Russia and Ukraine, and political knowledge, respectively – are interacted with the treatment indicators as well as the other covariates in the model. wer likelihood of believing and sharing fake news about the EU (Russia) and Tanzania.

	Dependent variable:	
	Fake News Beliefs	Fake News Sharing
	(1)	(2)
Russian Fake News (Rus. FN)	0.360*** (0.025)	0.174*** (0.029)
Tanzanian Fake News (Tan. FN)	0.226*** (0.026)	0.032 (0.029)
EU-vs-Ukraine Conflict Perceptions	0.259*** (0.047)	0.226*** (0.054)
Age	0.021* (0.011)	0.015 (0.013)
Education (1 = Low Edu.)	−0.026 (0.025)	0.007 (0.029)
Gender (1 = Male)	−0.005 (0.023)	0.029 (0.026)
Employment (1 = No work)	−0.030 (0.024)	−0.013 (0.027)
Political Knowledge	−0.015 (0.013)	−0.058*** (0.015)
Political Interest	0.020* (0.011)	0.041*** (0.013)
Ukrainian Identification	0.002 (0.013)	−0.025* (0.015)
Russian Power versus Ukraine	0.004 (0.011)	−0.001 (0.013)
European Identification	−0.026** (0.012)	−0.038*** (0.014)
Russian Identification	−0.002 (0.015)	−0.0001 (0.017)
EU Power versus Ukraine	−0.001 (0.012)	−0.012 (0.013)
Russia-vs-Ukraine Conflict Perceptions	0.040*** (0.011)	0.035*** (0.013)
EU-vs-Ukr Conflict Perc. X Rus FN	−0.495*** (0.050)	−0.287*** (0.057)
EU-vs-Ukr Conflict Perc. X Tan FN	−0.237*** (0.050)	−0.131** (0.057)
EU-vs-Ukr Conflict Perc. X Age	−0.035 (0.022)	−0.062** (0.025)
EU-vs-Ukr Conflict Perc. X Edu.	0.095** (0.047)	0.078 (0.054)
EU-vs-Ukr Conflict Perc. X Male	0.027 (0.043)	−0.023 (0.049)
EU-vs-Ukr Conflict Perc. X Empl.	0.051 (0.048)	0.037 (0.055)
EU-vs-Ukr Conflict Perc. X Pol. Know.	−0.004 (0.024)	0.042 (0.028)
EU-vs-Ukr Conflict Perc. X Pol. Int.	0.001 (0.021)	0.008 (0.024)
EU-vs-Ukr Conflict Perc. X Ukr. ID	0.001 (0.022)	0.037 (0.026)
EU-vs-Ukr Conflict Perc. X Rus. Power	0.006 (0.024)	−0.032 (0.027)
EU-vs-Ukr Conflict Perc. X EU ID	0.056** (0.022)	0.075*** (0.026)
EU-vs-Ukr Conflict Perc. X Rus. ID	0.001 (0.024)	0.031 (0.028)
EU-vs-Ukr Conflict Perc. X EU Power	−0.013 (0.025)	−0.007 (0.028)
EU-vs-Ukr Conflict Perc. X Rus-vs-Ukr Conflict Perc.	−0.031 (0.021)	−0.024 (0.024)
Intercept	0.174*** (0.023)	0.184*** (0.027)
Observations	1,605	1,605
Residual Std. Error (df = 1577)	0.230	0.266

Table SM2a. Effect of Treatment Conditions and Conflict Perceptions (Ukraine vs the EU) on Beliefs in and Intentions to Share Fake News. Regression coefficients with 95% confidence intervals in parentheses. Column to the left (right) shows results for beliefs in (intentions to share) fake news as the dependent variable. “Russian Fake News” and “Tanzanian Fake News” are treatment conditions (fake news about the EU are baseline condition). EU-vs-Ukraine conflict perceptions and the dependent variables have been scaled to range from 0 to 1. (see the main text for detailed discussion of the operationalizations). * $p < 0.10$, ** $p < 0.05$, *** $p < 0.01$.

	Dependent variable:	
	Fake News Beliefs	Fake News Sharing
	(1)	(2)
Russian Fake News (Rus. FN)	−0.265*** (0.062)	−0.301*** (0.070)
Tanzanian Fake News (Tan. FN)	−0.038 (0.058)	−0.182*** (0.066)
Ukrainian Identification	−0.137** (0.065)	−0.258*** (0.073)
Age	0.014 (0.026)	−0.032 (0.029)
Education (1 = Low Edu.)	0.098* (0.054)	0.034 (0.061)
Gender (1 = Male)	0.066 (0.052)	−0.001 (0.058)
Employment (1 = No work)	0.073 (0.060)	0.103 (0.068)
Political Knowledge	0.012 (0.027)	−0.030 (0.030)
Political Interest	−0.004 (0.027)	0.079*** (0.030)
Russian Power versus Ukraine	0.046 (0.031)	0.044 (0.035)
European Identification	0.003 (0.029)	0.015 (0.033)
Russian Identification	−0.031 (0.023)	−0.012 (0.026)
EU Power versus Ukraine	−0.012 (0.025)	−0.043 (0.029)
Russia-vs-Ukraine Conflict Perceptions	0.026 (0.028)	0.044 (0.031)
EU-vs-Ukraine Conflict Perceptions	0.026 (0.025)	−0.003 (0.028)
Ukrainian ID X Rus FN	0.477*** (0.069)	0.408*** (0.078)
Ukrainian ID X Tan FN	0.189*** (0.065)	0.187** (0.074)
Ukrainian ID X Age	−0.010 (0.029)	0.022 (0.032)
Ukrainian ID X Edu.	−0.095 (0.061)	0.012 (0.068)
Ukrainian ID X Male	−0.065 (0.057)	0.022 (0.065)
Ukrainian ID X Empl.	−0.089 (0.066)	−0.114 (0.075)
Ukrainian ID X Pol. Know.	−0.033 (0.030)	−0.009 (0.034)
Ukrainian ID X Pol. Int.	0.026 (0.030)	−0.040 (0.033)
Ukrainian ID X Rus. Power	−0.044 (0.033)	−0.064* (0.037)
Ukrainian ID X EU ID	−0.005 (0.032)	−0.020 (0.036)
Ukrainian ID X Rus. ID	0.032 (0.027)	0.030 (0.031)
Ukrainian ID X EU Power	0.011 (0.028)	0.036 (0.032)
Ukrainian ID X Rus-vs-Ukr Conflict Perc.	0.001 (0.030)	−0.021 (0.034)
Ukrainian ID X EU-vs-Ukr Conflict Perc.	−0.010 (0.028)	0.036 (0.031)
Intercept	0.399*** (0.059)	0.501*** (0.067)
Observations	1,605	1,605
Residual Std. Error (df = 1575)	0.234	0.265

Table SM2b. Effect of Treatment Conditions and Ukrainian Identification on Beliefs in and Intentions to Share Fake News. Regression coefficients with 95% confidence intervals in parentheses. Column to the left (right) shows results for beliefs in (intentions to share) fake news as the dependent variable. “Russian Fake News” and “Tanzanian Fake News” are treatment conditions (fake news about the EU are baseline condition). Ukrainian Identification and the dependent variables have been scaled to range from 0 to 1. (see the main text for detailed discussion of the operationalizations). * $p < 0.10$, ** $p < 0.05$, *** $p < 0.01$.

	Dependent variable:	
	Fake News Beliefs	Fake News Sharing
	(1)	(2)
Russian Fake News (Rus. FN)	0.337*** (0.033)	0.199*** (0.038)
Tanzanian Fake News (Tan. FN)	0.178*** (0.032)	0.073** (0.037)
Russian Power versus Ukraine	0.161*** (0.042)	0.065 (0.047)
Age	0.021 (0.014)	−0.010 (0.016)
Education (1 = Low Edu.)	−0.012 (0.030)	0.018 (0.034)
Gender (1 = Male)	0.016 (0.029)	−0.005 (0.033)
Employment (1 = No work)	0.058* (0.030)	0.038 (0.034)
Political Knowledge	−0.027* (0.014)	−0.046*** (0.016)
Political Interest	0.012 (0.014)	0.047*** (0.016)
Ukrainian Identification	0.009 (0.016)	0.006 (0.018)
European Identification	−0.005 (0.015)	−0.007 (0.017)
Russian Identification	0.004 (0.019)	0.045** (0.022)
EU Power versus Ukraine	0.003 (0.011)	−0.017 (0.013)
Russia-vs-Ukraine Conflict Perceptions	0.038*** (0.013)	0.045*** (0.015)
EU-vs-Ukraine Conflict Perceptions	0.025* (0.015)	0.055*** (0.017)
Rus. Power X Rus FN	−0.281*** (0.046)	−0.221*** (0.052)
Rus. Power X Tan FN	−0.079* (0.045)	−0.149*** (0.052)
Rus. Power X Age	−0.029 (0.020)	−0.010 (0.023)
Rus. Power X Edu.	0.050 (0.043)	0.042 (0.049)
Rus. Power X Male	−0.016 (0.040)	0.032 (0.045)
Rus. Power X Empl.	−0.100** (0.043)	−0.051 (0.049)
Rus. Power X Pol. Know.	0.011 (0.020)	0.008 (0.023)
Rus. Power X Pol. Int.	0.016 (0.019)	−0.001 (0.022)
Rus. Power X Ukr. ID	−0.011 (0.021)	−0.018 (0.023)
Rus. Power X EU ID	0.008 (0.021)	0.008 (0.023)
Rus. Power X Rus. ID	−0.009 (0.024)	−0.041 (0.027)
Rus. Power X EU Power	−0.012 (0.020)	0.007 (0.022)
Rus. Power X Rus-vs-Ukr Conflict Perc.	−0.023 (0.019)	−0.038* (0.021)
Rus. Power X EU-vs-Ukr Conflict Perc.	−0.014 (0.020)	−0.041* (0.023)
Intercept	0.180*** (0.030)	0.236*** (0.034)
Observations	1,605	1,605
Residual Std. Error (df = 1575)	0.234	0.265

Table SM2c. Effect of Treatment Conditions and Russian Power vs Ukraine on Beliefs in and Intentions to Share Fake News. Regression coefficients with 95% confidence intervals in parentheses. Column to the left (right) shows results for beliefs in (intentions to share) fake news as the dependent variable. "Russian Fake News" and "Tanzanian Fake News" are treatment conditions (fake news about the EU are baseline condition). Russian Power vs Ukraine and the dependent variables have been scaled to range from 0 to 1. (see the main text for detailed discussion of the operationalizations). * $p < 0.10$, ** $p < 0.05$, *** $p < 0.01$.

	Dependent variable:	
	Fake News Beliefs	Fake News Sharing
	(1)	(2)
Russian Fake News (Rus. FN)	0.106** (0.053)	0.031 (0.059)
Tanzanian Fake News (Tan. FN)	0.105** (0.051)	0.031 (0.057)
Political Knowledge	−0.165** (0.076)	−0.240*** (0.085)
Age	0.028 (0.023)	−0.035 (0.026)
Education (1 = Low Edu.)	−0.010 (0.045)	0.041 (0.051)
Gender (1 = Male)	0.054 (0.046)	0.035 (0.051)
Employment (1 = No work)	−0.082* (0.048)	−0.071 (0.053)
Russian Power versus Ukraine	0.005 (0.024)	−0.021 (0.027)
Political Interest	0.051** (0.022)	0.102*** (0.025)
Ukrainian Identification	0.008 (0.022)	−0.029 (0.024)
European Identification	−0.015 (0.024)	0.0002 (0.027)
Russian Identification	0.002 (0.024)	0.011 (0.027)
EU Power versus Ukraine	−0.006 (0.022)	0.006 (0.025)
Russia-vs-Ukraine Conflict Perceptions	0.061*** (0.023)	0.094*** (0.026)
EU-vs-Ukraine Conflict Perceptions	0.021 (0.024)	−0.002 (0.027)
Pol. Know. X Rus FN	0.075 (0.082)	0.039 (0.092)
Pol. Know. X Tan FN	0.033 (0.080)	−0.086 (0.090)
Pol. Know. X Age	−0.042 (0.036)	0.032 (0.041)
Pol. Know. X Edu.	0.043 (0.073)	0.001 (0.082)
Pol. Know. X Male	−0.069 (0.072)	−0.019 (0.081)
Pol. Know. X Empl.	0.129* (0.075)	0.128 (0.085)
Pol. Know. X Rus. Power	0.0001 (0.038)	0.012 (0.043)
Pol. Know. X Pol. Int.	−0.049 (0.035)	−0.097** (0.040)
Pol. Know. X Ukr. ID	−0.010 (0.035)	0.039 (0.039)
Pol. Know. X EU ID	0.022 (0.039)	−0.006 (0.044)
Pol. Know. X Rus. ID	−0.009 (0.038)	0.003 (0.043)
Pol. Know. X EU Power	0.009 (0.037)	−0.032 (0.042)
Pol. Know. X Rus-vs-Ukr Conflict Perc.	−0.061* (0.037)	−0.117*** (0.041)
Pol. Know. X EU-vs-Ukr Conflict Perc.	−0.011 (0.037)	0.043 (0.042)
Intercept	0.387*** (0.048)	0.423*** (0.054)
Observations	1,605	1,605
Residual Std. Error (df = 1575)	0.237	0.266

Table SM2d. Effect of Treatment Conditions and Political Knowledge on Beliefs in and Intentions to Share Fake News. Regression coefficients with 95% confidence intervals in parentheses. Column to the left (right) shows results for beliefs in (intentions to share) fake news as the dependent variable. “Russian Fake News” and “Tanzanian Fake News” are treatment conditions (fake news about the EU are baseline condition). Political Knowledge and the dependent variables have been scaled to range from 0 to 1. (see the main text for detailed discussion of the operationalizations). * $p < 0.10$, ** $p < 0.05$, *** $p < 0.01$
